# Single-cell technologies are revolutionizing the approach to rare cells

**DOI:** 10.1038/icb.2015.106

**Published:** 2015-12-22

**Authors:** Valentina Proserpio, Tapio Lönnberg

**Affiliations:** 1 Wellcome Trust Sanger Institute, Wellcome Trust Genome Campus, Cambridge, UK; 2 European Molecular Biology Laboratory-European Bioinformatics Institute (EMBL-EBI), Wellcome Trust Genome Campus, Cambridge, UK

## Abstract

In the last lustrum single-cell techniques such as single-cell quantitative PCR, RNA and DNA sequencing, and the state-of-the-art cytometry by time of flight (CyTOF) mass cytometer have allowed a detailed analysis of the sub-composition of different organs from the bone marrow hematopoietic compartment to the brain. These fine-grained analyses have highlighted the great heterogeneity within each cell compartment revealing previously unknown subpopulations of cells. In this review, we analyze how this fast technological evolution has improved our understanding of the biological processes with a particular focus on rare cells of the immune system.

## Introduction

We consider a human being as a single being, unique and unrepeatable. However, in the last few years multiple single-cell technologies have revealed that we are not only unique and unrepeatable, but we might be composed of millions of millions of unique and unrepeatable cells.^1, 2, 3, 4, 5^ Most of our scientific knowledge originates from population data where cells belonging to the same subtype are considered as a single unit, in which all the members that compose the class are, by definition, homogeneous and identical. This concept represents an extreme simplification of the reality and can be ascribed both to the necessary simplification required to understand the global picture and also to the lack of technologies and data analysis techniques that allow more fine-grained investigations.

The absence of technologies for studying single cells has had the greatest impact on the analysis of cells that are occur as a minimal fraction of the initial population: rare cells.

A cell is considered ‘rare' when the number of cells of that particular subpopulation represent a fraction of the total heterogeneous population <0.01%. Examples of rare cells are circulating tumour cells (CTCs)^6^ and circulating fetal cells in the peripheral blood.^7^ More specifically for the immune system, rare cells include antigen-specific lymphocytes and hematopoietic stem cells (HSC).^8^

In the immune context, capturing and analyzing rare cells in a heterogeneous population is of vital importance especially for studying key regulatory pathways both in the steady-state and during disease progression.^9^

So far, the instrument that has allowed most of the study of rare cell is the flow cytometer, where up to 17 different phenotypic markers can be analyzed at the same time per cell.^10^ The most sophisticated and recent machine, with a flow rate of up to 100 000 events per second, allows the detection of cells at frequencies as low as 0.0001%. The combination of this technology with the parallel development of both new specific fluorophore-conjugated antibodies, which presently span the whole visible and part of the near-infrared spectrum, and also better and faster analysis algorithms is key for the identification and isolation of rare cells.

**Figure 1:**
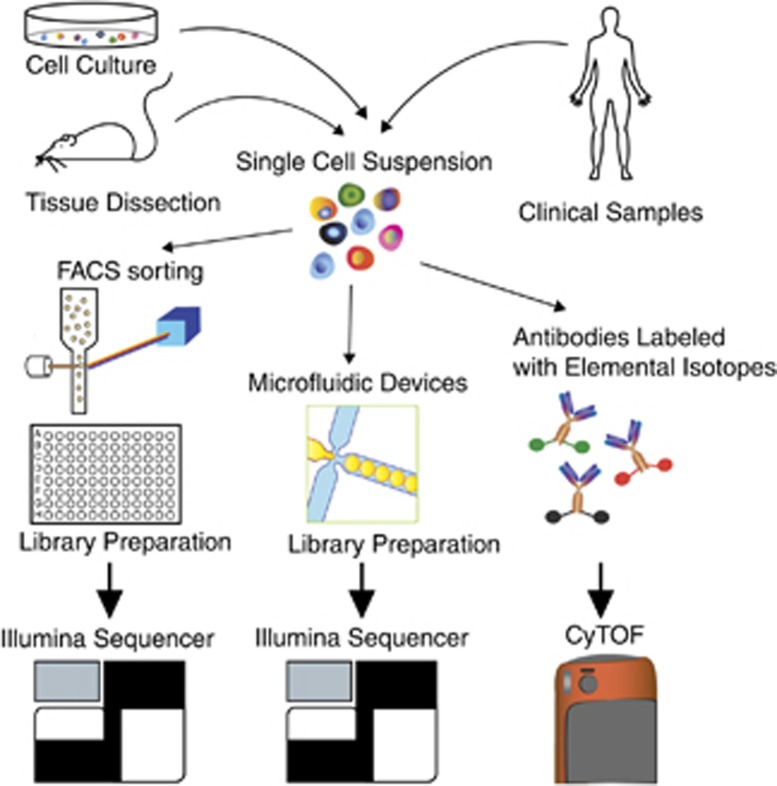
Schematic diagram of different single-cell techniques. First, a single-cell suspension can be obtained from animal tissues, cultured cells and from clinical samples. For genome-wide transcriptome profiling, cells of interest can be FACS-sorted into multiwell (96 or 384) plates, and library preparation can be performed manually or using a liquid-handling robot (on the left). Cell capturing and library preparation can alternatively be performed using microfluidic devices (middle panel). Single-cell proteomics by mass cytometry can be performed with the cytometry by time of flight (on the right). First, cells are stained with antibodies coupled to isotopically purified mass tags and then analyzed through a mass spectrometer. The individual ions are counted and then resolved into a flow cytometry file format. With this technique, about 40 simultaneous antigens can be quantified in individual cells at a rate of about 500-1000 cells per s. FACS, fluorescence-activated cell sorting.

In the last few years an abundance of different single-cell technologies has been developed that allows even higher-dimensional analyses of isolated single cells (Figure 1). Single-cell quantitative PCR,^11^ can quantify the expression of hundreds of genes by an adaptation of standard quantitative real-time PCR. Single-cell proteomic analysis with cytometry by time of flight (single-cell mass cytometry)^12^ currently allows the detection of the levels of up to 40 different proteins within the same cell. Finally, single-cell DNA and RNA sequencing methods can determine entire transcriptomic and genomic profiles.^1, 13, 14, 15, 16, 17, 18, 19^

Single-cell sequencing was, in 2013, appointed by the journal *Nature Methods* as the method of the year as it allows a comprehensive view of both the transcription profile and the DNA content in one cell without requiring *a priori* knowledge of genes of interest.

So far, single-cell sequencing has allowed scientists to characterize rare cells such as single neurons,^20^ circulating tumour cells^21^ and individual sperm cells.^22^

This global and unbiased approach will not only shed more light on the behaviour of rare cells, but will also increase the number of different rare cell types that have been described.

## Newly identified rare subpopulations in the immune system

Single-cell sequencing has shown that almost each cell is different to every other cell^23^ and has identified previously unknown hidden subpopulation of cells within the populations under study.

Employing single-cell RNA sequencing on both *in vivo* and *ex vivo* cultured T helper (Th) cells, Mahata *et al.*^24^ identified a subpopulation of cells that produce the steroid pregnenolone within the Th2 subtype. This steroid production was linked with the presence of the enzyme Cyp11a1 and was shown to suppress T-cell proliferation. This new Th2 subset, thought to contribute to the maintenance of the homoeostasis, would have not been identified with classical measurements of bulk populations, as it would have been masked by the other more prevalent cells.

In the same way, Shalek *et al.* found a very small subset of cells that they named precocious expressers among mouse bone marrow (BM)-derived dendritic cells.^23^ Those particular cells are the ones that, in response to lipopolysaccharide-induced inflammatory stimulus, first produce and secrete a wave of interferon in order to coordinate a gradual core antiviral response. The activity of those cells was also tested in the absence of cell-to-cell communication by stimulation of cells after their capture within a microfluidic chamber.

Recently, high-dimensional mass cytometry (cytometry by time of flight) was integrated with phospho-specific flow cytometry and novel computational data analysis to study the heterogeneity of the ROS-mediated regulation of B-cell receptor signalling within subsets of primary human tonsillar B cells.^25^ This allowed the identification of a small subpopulation of H_2_O_2_ responsive cells and determined their identity with a mass cytometry panel designed to characterize mature B cells. The so called ‘responder' cell phenotype suggested a germinal centre B-cell identity.

Overall, these findings well-represent the potential of the single-cell research, as those specific subsets of cells would have not been identified with traditional population analysis.

The impact of such research can be extremely important, especially in immunology where cell-to-cell variation is notoriously high and is key to the success of the immune response.

## Antigen-specific T cell clones

Tremendous cellular diversity originates from the somatic rearrangement of T-cell receptor (*TCR*) genes during their development in the thymus. Proposed theoretical numbers of possible distinct human TCRs are as high as 10^15^ or 10^16^ ^26, 27^ and a single individual has been estimated to have in the order of 10^8^ T-cell clones.^27^ Approximately only one in every 10^6^ to 10^7^ naive human T cells are able to react to a given antigen epitope, highlighting the immense functional specialization.^28^ Thus the T-cell repertoire contains enormous diversity, as well as flexibility, for alterations of this repertoire in face of changing conditions. Decreased TCR diversity can result from various disease states, such as systemic lupus erythematosus.^29^ Typically, the T-cell repertoire also becomes somewhat narrower during old age accompanied by changes in clonal frequencies, reduced immune function and diminished response to vaccinations.^30^ While this decline in immune function is not completely understood, it is likely to result from a combination of thymic involution and infections by latent pathogens such as cytomegalovirus.^31^

To date, the exact relationship of TCR sequence and functional potential of the cell remains to be conclusively elucidated. Progeny of antigen-specific CD4+ T cells purified using peptide-major histocompatibility complex-multimer staining and cell sorting are able to assume multiple alternative cell fates (Th1, Th2 and Th17) *in vitro*.^32^ *In vivo*, CD4+ T cells with transgenic recombinant TCRs have been shown to differentiate to both Th1 and Tfh-type cells, with the frequencies influenced by the strength of TCR signalling.^33^ With such findings, it is obvious that the functional potential of a T-cell clone is not restricted by its TCR sequence. Nevertheless, it is less clear how the receptor structure influences the clone's propensity for alternative cell fates *in vivo*.

Recently, powerful tools for characterizing antigen-specific T-cell populations have been developed. Possibilities of conventional flow cytometry-based assays have been extended with mass cytometry technology, which has been used in conjunction with peptide-major histocompatibility complex-multimer staining on CD8+ T cells by Newell *et al.*^34^ Using a panel of 77 rotavirus-specific peptide-tetramers tagged in a combinatorial fashion, coupled with a set of 27 antibodies against functional marker proteins, it was possible to identify T cells reactive against different rotavirus epitopes in distinct regulatory states.

However, as cytometry experiments inevitably consume the sample, simultaneous *de novo* determination of TCR sequence is not possible. A strategy for obtaining TCR sequence and functional information from the same single cells, was recently published by Han *et al.*^9^ Using nested PCR amplification of the TCR, in combination with assays for 17 phenotyping markers, the authors could at the same time determine the exact TCR and achieve a rough functional classification of the measured cells. Recently, completely unbiased analysis of functional state in combination with TCR sequencing has become possible with the advent of single-cell RNA sequencing. We have developed a computational strategy for reconstructing TCR sequences from genome-wide single-cell transcriptomics data (Stubbington *et al.*) and applied it to murine CD4+ T cells activated during a *Salmonella typhimurium* infection. Our results demonstrated significant clonal expansion during the infection, and indicated that cells of a single T-cell clone can populate distinct functional compartments (Th1 effector, central memory and effector memory) at the same time.

## Heterogeneity of the cells of the thymic medulla

Functionally divergent populations of rare cells can also arise as a result of the stochastic action of genes that are expressed at similar levels across the cells. An example of such process is seen in the epithelial cells of the thymic medulla (mTEC). These cells are crucial in purging autoreactive T cells and promoting development of thymic regulatory T cells by ‘projecting an immunological self shadow' through systematic expression of tissue-restricted antigens (TRA). In mTECs, the^35^ expression of the *Aire* gene, coding for autoimmune regulator protein, activates transcription of a large number of genes in an apparently stochastic, sequence-independent manner, and its deletion results in severe autoimmunity.^35^

Recent studies exploiting the potential of single-cell sequencing have for the first time systematically delineated the action of AIRE in mTECs. Brennecke *et al.*^36^ have shown that while each of the *Aire*-induced genes is expressed by only a small fraction of the mTEC cells, at the population level 11 distinct co-expression clusters of these *TRA* genes can be detected, indicating a previously unknown level of organization in this seemingly random process. Furthermore, their data together with results of previous targeted assays^37^ suggested that the mTECs would transition from one of the 11 co-expression clusters to the next in a staged and directional manner. Both Brennecke *et al.* and a back-to-back publication by Meredith *et al.*^38^suggested that the expression of the *Aire*-dependent *TRA* genes is enriched in chromosomal microclusters that are scattered across the genome. Notably, Meredith *et al.* further demonstrated that these microclusters are specific to individuals and shared by multiple single cells. Since the genes in these clusters did not share detectable regulatory motifs, the most plausible explanation for this is a common developmental origin, that is, the cells with shared TRA clusters represent daughters of the same progenitor that underwent an early heritable AIRE-induced activation of the *TRA* genes.^38^ In light of these findings, it is justifiable to consider the thymic epithelium as a mosaic of rare, functionally specialized, but developmentally related subpopulations of the mTECs.

## Haematopoietic stem cells

HSC have the ability to self-renewal and to differentiate into all the other blood cells. They derive from the mesoderm and reside primarily in the BM.^39^ BM-HSCs represent only a small fraction of total BM cells, accounting for roughly 0.01 % of the total (Table 1).

Haematopoiesis is one of the most studied differentiation process both because of the broad availability of those cells and because of the existence of a discrete hierarchy of differentiation stages.^40^ Even though it has been widely studied, HSC cells have been erroneously considered for many years a homogenous population of cells with flexible behaviour. In the last decade, a completely different picture has emerged and HSCs are now known to be characterized by a great level of heterogeneity^41, 42^ in multiple aspects, including their proliferative and self-renewal potential,^43^ lifespan and also a differentiation preference towards the lymphoid lineage or the myeloid lineage.^44^

For all these reasons single-cell sequencing has been employed in the past few years in order to shed more light on this intrinsic heterogeneity.

Tsang *et al.*^45^ profiled the transcriptomes of 180 cells in the mouse adult HSC compartment and studied the expression profile structure and the role of *Bcl11a*. First, they highlight how cell cycle fluctuation is the main source of transcriptional variation in the HSC compartment. Second, Bcl11a^−/−^ transcriptomic analysis revealed abnormal proliferative phenotypes with reduced quiescence, reduced self-renewal potential and higher proliferation, which was experimentally confirmed by BrdU staining. This study demonstrates again the power of single-cell transcriptomics in dissecting cellular process and lineage heterogeneities in stem cell compartments.

Wilson *et al.*^46^ employed single-cell index sorting coupled with sequencing to analyze the molecular signature of HSC sorted with four different purification strategies. This allowed the construction of a common signature gene list that can be use to single separate out the most likely functional HSCs.

High-throughput quantitative real-time PCR on single primary blood stem cell and BM-HSC during early haematopoietic differentiation stages have also revealed that different populations are characterized by related, but distinctive transcription factor expression states.^47^

Another study on early blood cell development at single-cell resolution has focused on transcription factors as master regulators of cell fate.^48^ Using quantitative reverse transcription-PCR, Moignard and co-workers analyzed 46 genes in about 4000 cells from 5 populations at 4 sequential stages of post-implantation mouse development between embryonic day 7.0 and 8.25. They combined the gene expression profiles with an elegant computational approach based on diffusion maps for dimensionality reduction and network synthesis from state transition graphs in order to determine the structure and function of transcriptional regulatory networks. The analysis showed that cellular maturation might be asynchronous, with individual cells maturing at different speeds. This study demonstrates how network modelling from single cells can help to reveal the transcriptional hierarchies that control mammalian development.

From the proteomic point of view, Bendall *et al.*^49^ combined the single-cell mass cytometry with an algorithm, named Wanderlust, to align single cells of a given lineage onto a hierarchical trajectory, thus predicting the developmental path. This approach allowed them to perform an in-depth examination of rare and transient early stages of B-cell development and identify previously unknown populations of B-cell progenitors based on the combined expression of CD34, CD38, CD24 and TdT.

## Future application of single-cell technologies on blood-derived HSC

A very small percentage of HSC cells are able to leave the BM and circulate into the blood stream. These cells are called peripheral blood stem cells.^50^ Despite their extreme low frequency, *ca.* 1 per million nucleated cells, these circulating progenitor cells with clonogenic properties have been of great interest because of their ability to substitute BM-derived HSC in transplants to treat patients with cancers and other disorders of the blood and immune systems.^51, 52^ Moreover, some studies reported that peripheral blood stem cell are not only able to differentiate into all the hematopoietic lineages, but are also able to differentiate into other cell lineages, such as adipocytes,^53^ thus suggesting that these cells hold great potential for applications in tissue engineering outside the hematopoietic system.

Unfortunately, an adequate peripheral blood stem cell collection is a pre-requisite for most of their clinical applications.

Those cells are known to be extremely heterogeneous but, due to their extreme low abundance, their heterogeneity has not been yet properly investigated.

All these features make the new single-cell technologies extremely valuable to obtain a more comprehensive view of these cells and to analyze their heterogeneity.

Initially, the identification of new specific molecular targets for improving the mobilization of peripheral blood stem cells from the BM would be crucial for increasing the success rate in clinical applications as in advanced haematological malignancies.

## Concluding remarks

In the immune context, bulk measurements have contributed to most of our current knowledge of disease pathogenesis and immunity, but they failed to report a detailed picture of the players. Cell-to-cell variation, gene co-regulation and transcriptional networks cannot be precisely inferred by population analysis. In the immune system, where cells are known to display a very high level of heterogeneity, a deeper analysis of the gene expression fluctuations is of extreme importance with broad implications for both basic and clinical research. Despite the great progress that has been made in the past few years in the single-cell technology field, further developments of those techniques will be extremely beneficial in order to obtain a more fine-grained picture of all the molecular events leading to pathogenesis of morbid conditions or of disease. In particular, the ability of sequencing non polyadenylated RNA and both microRNA and small RNA, the integration of the transcriptomic profile with cell position in three dimensions, the standardization of both the experimental procedure and the computational analysis of the single-cell profiling will be the next crucial steps for enabling a deeper analysis of both the steady-state and the disease mechanisms.

## Figures and Tables

**Table 1:** Examples and frequency of rare cell types

*Cell type*	*Frequency*
Antigen-specific T cells	1 cell per 10^6^ cells (in peripheral blood)
Circulating tumour cells	1 cell per 10^7^ cells (in peripheral blood)
Endothelial progenitor cells	1 cell per 10^5^ cells (in peripheral blood)
Hematopoietic stem cells	1 cell per 10^4^ cells (in bone marrow)
Hematopoietic stem cells	1 cell per 10^6^ cells (in peripheral blood)
